# Altered metabolic connectivity within the limbic cortico-striato-thalamo-cortical circuit in presymptomatic and symptomatic behavioral variant frontotemporal dementia

**DOI:** 10.1186/s13195-022-01157-7

**Published:** 2023-01-05

**Authors:** Li Liu, Min Chu, Binbin Nie, Deming Jiang, Kexin Xie, Yue Cui, Lin Liu, Yu Kong, Zhongyun Chen, Haitian Nan, Pedro Rosa-Neto, Liyong Wu

**Affiliations:** 1 Department of Neurology, Xuanwu Hospital, Capital Medical University, Changchun Street 45, Beijing, 100053 China; 2 Beijing Engineering Research Center of Radiographic Techniques and Equipment, Institute of High Energy Physics, Chinese Academy of Sciences, Beijing, China; 3 School of Nuclear Science and Technology, University of Chinese Academy of Sciences, Beijing, China; 4 Department of Neurology, Second Hospital of Shanxi Medical University, Taiyuan, China; 5 McGill Centre for Studies in Aging, Alzheimer’s Disease Research Unit, Montreal, H4H 1R3 Canada

**Keywords:** Frontotemporal dementia, *MAPT* gene, Metabolic connectivity, Striatum, Cortico-striato-thalamic-cortical (CSTC) circuit

## Abstract

**Background:**

Behavioral variant frontotemporal dementia (bvFTD) is predominantly considered a dysfunction in cortico-cortical transmission, with limited direct investigation of cortical-subcortical transmission. Thus, we aimed to characterize the metabolic connectivity between areas of the limbic cortico-striato-thalamic-cortical (CSTC) circuit in presymptomatic and symptomatic bvFTD patients.

**Methods:**

Thirty-three bvFTD patients and 33 unrelated healthy controls were recruited for this study. Additionally, six asymptomatic carriers of the *MAPT* P301L mutation were compared with 12 non-carriers who were all from the same family of bvFTD. Each participant underwent neuropsychological assessment, genetic testing, and a hybrid PET/MRI scan. Seed-based metabolic connectivity based on [^18^F]-fluorodeoxyglucose PET between the main components within the limbic CSTC circuit was explored according to the Oxford-GSK-Imanova Striatal Connectivity Atlas.

**Results:**

BvFTD patients exhibited reduced metabolic connectivity between the relays in the limbic CSTC circuit, which included the frontal region (ventromedial prefrontal cortex, orbitofrontal cortex, rectus gyrus, and anterior cingulate cortex), the limbic striatum, and thalamus compared to controls. In the bvFTD patients, the involvement of the limbic CSTC circuit was associated with the severity of behavior disruption, as measured by the frontal behavior inventory, the disinhibition subscale, and the apathy subscale. Notably, asymptomatic *MAPT* carriers had weakened frontostriatal connectivity but enhanced striatothalamus and thalamofrontal connectivity within the limbic CSTC circuit compared with noncarriers.

**Conclusion:**

These findings suggested that aberrant metabolic connectivity within the limbic CSTC circuit is present in symptomatic and even asymptomatic stages of bvFTD. Thus, metabolic connectivity patterns could be used as a potential biomarker to detect the presymptomatic stage and track disease progression.

**Supplementary Information:**

The online version contains supplementary material available at 10.1186/s13195-022-01157-7.

## Background

Behavioral variant frontotemporal dementia (bvFTD) is a highly genetic and heterogeneous clinical syndrome characterized by early and prominent deterioration of behavior, which includes impaired social interactions, disinhibition, apathy, and impairment in adaptive functioning, and the main causal genes are mutations of the microtubule-associated protein tau (MAPT) gene, especially in China [1–3]. Asymptomatic carriers with autosomal dominant mutations provide an opportunity to investigate the pathophysiology of bvFTD in the earliest phases of the illness [4]. Although bvFTD is traditionally considered a frontotemporal cortical disease, there is growing evidence that subcortical brain regions, particularly the striatum, are also significantly affected and may play a role in the generation of motor, cognitive, behavioral, and psychiatric symptoms [5–7]. However, limited attention has been drawn toward the striatum, especially for the limbic striatum, which is specifically associated with the central features of bvFTD: behavioral and psychiatric disturbances [8, 9].

Given that the dysfunction is not solely attributable to the nature of isolated regions, neural circuits mediated by the limbic striatum are critical for the integration of information to carry out goal-directed behavior and psychiatric function [10]. The limbic striatum receives projections from the prefrontal cortex, which includes the ventromedial prefrontal cortex (vmPFC), orbitofrontal cortex (OFC), rectus gyrus, and anterior cingulate cortex (ACC), and sends projections that ultimately relay information back to the prefrontal cortex via the thalamus, which constitutes the limbic CSTC circuit [11, 12]. Multiple lines of evidence in patients with different diseases suggested that the dysfunction of this limbic CSTC circuit plays a major role in the pathogenesis of behavioral and psychiatric symptoms, with the limbic striatum serving as the key subcortical relay [9, 13, 14]. Previous neuroimaging studies have revealed that the behavioral and psychiatric disturbances in patients with bvFTD may be attributed to structural abnormalities of the relays within the limbic CSTC circuits, which includes the vmPFC, OFC, rectus gyrus, ACC, and especially the limbic striatum; moreover, these abnormalities can be detected even in asymptomatic mutation carriers [15–18]. Recently, the alteration in the white matter microstructure of the OFC and limbic striatum connections were detected in bvFTD patients using diffusion tensor imaging (DTI) [5]. These changes indicate a possible role of the limbic CSTC circuit in the genesis of the neuropsychiatric symptoms in bvFTD. However, to date, there is fragmentary evidence of altered limbic CSTC circuit structure and integrity in bvFTD patients, and whether abnormalities in the connectivity between the brain regions are present remains unclear. In particular, the specific contributions of the limbic CSTC circuit to the behavioral and psychiatric abnormalities of bvFTD need to be clarified. We hypothesized that aberrant metabolic connectivity within the limbic CSTC circuit is present in bvFTD patients, and plays a role in the core manifestations, such as behavioral and psychiatric symptoms.

In the current study, we examined metabolic connectivity-based [^18^F]-fluorodeoxyglucose (FDG) PET/MRI data within the limbic CSTC circuits in bvFTD patients and asymptomatic *MAPT* carriers. In addition, we explored associations between FDG standardized uptake value ratio (SUVR) in the main relays of the CSTC circuit and symptom severity in the bvFTD group, with a particular focus on the limbic striatum. We aimed to explore the characteristics of the involvement of the limbic CSTC circuit in the continuous spectrum of bvFTD, as well as its relationship with behavioral disturbance.

## Methods

### Participants

We enrolled 33 bvFTD patients from July 1, 2017, to December 31, 2020, at the Department of Neurology of Xuanwu Hospital, who fulfilled the 2011 consensus probable bvFTD criteria [19]. We also recruited 33 age- and sex-matched healthy individuals, who were unrelated to the patients, to serve as the controls for bvFTD patients. All participants underwent clinical interviews, physical examinations, neuropsychological assessments, genetic testing, and a brain [^18^F]-FDG PET/MRI.

In addition, 18 asymptomatic participants were recruited from the Department of Neurology of Xuanwu Hospital in September 2017, who belonged to a family with an autosomal dominant P301L mutation of the *MAPT* gene. We defined participants as asymptomatic when both they and their spouse denied cognitive and behavioral disturbances and had normal scores on neuropsychiatric measures. All participants underwent genetic screening, and six participants were found to be carriers of the mutation. The remaining 12 were mutation-negative and were used as controls owing to their similar early environment, genetic background, and demographics. Each participant underwent clinical interviews, physical examinations, neuropsychological assessments, and a brain [^18^F]-FDG PET/MRI. All participants had been followed up prospectively with annual clinical examinations between September 2017 and October 2021 at Xuanwu Hospital. During the 4-year follow-up period, all subjects remained symptom-free and none developed any bvFTD symptoms or any other neurodegenerative disease.

### Neuropsychological assessments

The neuropsychological test battery consisted of widely used neuropsychological assessments that measure the cognitive function in the domains of memory, language, and behavioral abnormalities. Global cognitive screening measures comprised the Mini-Mental State Examination (MMSE), the Montreal Cognitive Assessment (MoCA), and the Frontotemporal Lobar Degeneration-Clinical Dementia Rating scale (FTLD-CDR). Word-list memory was evaluated using Rey’s Auditory-Verbal Learning Test (AVLT). Language was evaluated using the Boston Naming Test (BNT). The severity of behavioral abnormalities was assessed using the Frontal Behavior Inventory (FBI), which is divided into the negative apathy symptom subscale (first 12 items) and the positive disinhibition symptom subscale (last 12 items).

### PET/MRI acquisition parameters

All images were acquired on a hybrid 3.0 T TOF PET/MRI scanner (SIGNA PET/MR, GE Healthcare, WI, USA) [20]. PET and MRI data were acquired simultaneously using a vendor-supplied 19-channel head and neck union coil. Subjects were injected intravenously with [^l8^F]-FDG (3.7 MBq/kg), and underwent three-dimensional (3D) T1-weighted sagittal imaging and [^l8^F]-FDG-PET imaging 40 min later during the same session.

A 3D T1-weighted fast field echo sequence (repetition time [TR] = 6.9 ms, echo time [TE] = 2.98 ms, flip angle = 12°, inversion time = 450 ms, matrix size = 256 × 256, field of view = 256 × 256 mm^2^, slice thickness = 1 mm, 192 sagittal slices with no gap, voxel size = 1 × 1 × 1 mm3, and acquisition time = 4 min 48 s) was used for data acquisition. Static [^l8^F]-FDG-PET data were acquired using the following scanning parameters: matrix size = 192 × 192, field of view = 350 × 350 mm^2^, and pixel size = 1.82 × 1.82 × 2.78 mm3, and included corrections for random coincidences, dead time, scatter, and photon attenuation.

### Structural image preprocessing

Data were preprocessed using the Computational Anatomy Toolbox (CAT12) toolbox segment data pipeline implemented within Statistical Parametric Mapping 12 (SPM12, www.fl.ion.ucl.ac.uk/spm). Structural MRI images were normalized to standard Montreal Neurological Institute (MNI) space using diffeomorphic anatomical registration through exponentiated lie algebra normalization as implemented in SPM12. The images were then smoothed using an 8-mm full-width half-maximum isotropic Gaussian kernel for all directions.

### PET/MRI image preprocessing

The [^l8^F]-FDG PET image processing and analyses were performed using SPM12 implemented in the Matlab software (Mathwork, Inc., Natick, MA, USA). After normalizing the structural MRI images, the transformation parameters determined by the T1-weighted image spatial normalization were applied to the co-registered PET images for PET spatial normalization. The images were then smoothed using an isotropic Gaussian kernel with an 8-mm full-width half-maximum. The FDG-PET scan intensity was normalized using a whole cerebellum reference region to generate standardized uptake value ratio (SUVR) images.

### Analysis at the striatal subregion level

**Fig. 1:**
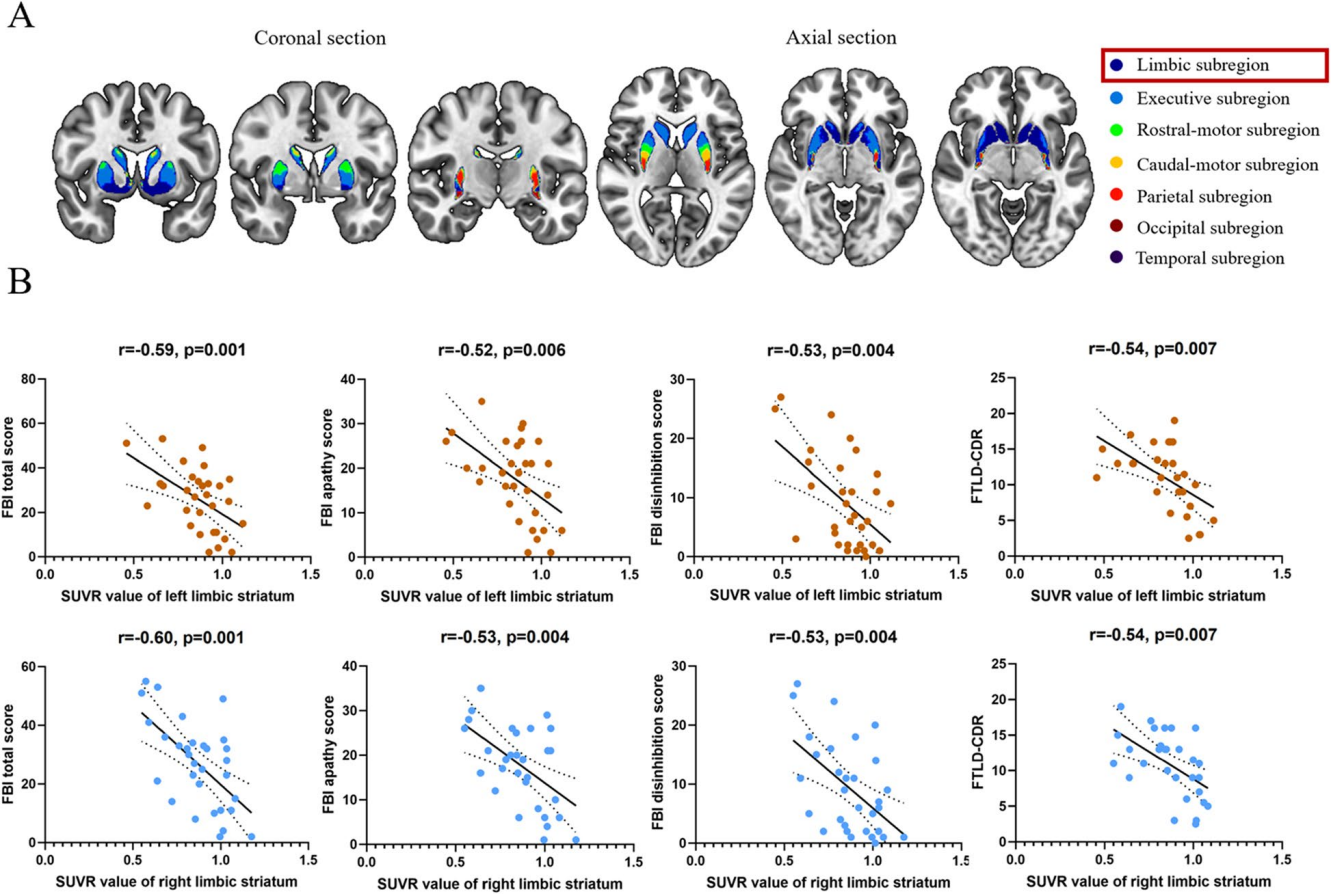
Significant correlations between the limbic striatum and neuropsychological scores in patients with bvFTD. **A** Striatal parcellations based on intrinsic functional connectivity with the cerebral cortex. **B** Scatter plots of significant correlations between the SUVR value of the limbic striatum and neuropsychiatric scores. Region and scatterplot colors, red: left limbic striatum, blue: right limbic striatum

We used the substriatal regions of interest (ROIs) from the Oxford-GSK-Imanova Striatal Connectivity Atlas [9], which is a probabilistic atlas of substriatal regions segmented according to their white-matter connectivity to cortical regions. Based on the differential cortical connectivity patterns, the atlas subdivides the striatum into seven subregions: limbic, executive, rostral-motor, caudal-motor, parietal, occipital, and temporal subregions (Fig. 1A). We focused on the limbic striatum because it is the principal subcortical relay of the limbic CSTC circuits. Mean [^18^F]-FDG PET SUVRs were determined separately in the unilateral limbic portion of the striatum using ROIs provided by the atlas.

### Metabolic connectivity analysis of the limbic CSTC circuit

We used sparse inverse covariance estimation (SICE), which is a method previously validated by Huang et al. [21] A series of nodes (*N* = 172) that represent brain ROIs for the connectivity analysis were selected to cover the whole brain [22, 23]. Because our hypothesis was specifically focused on the limbic CSTC circuit, we selected six ROIs for the functional connectivity analysis, which included the vmPFC, OFC, rectus gyrus, ACC, limbic striatum, and thalamus (i.e., the main components of the limbic CTSC circuitry). We performed a seed-based analysis with the six ROIs to investigate the connectivity between the frontal cortex and the limbic striatum, the limbic striatum and the thalamus, and the thalamus and the frontal cortex.

### Statistical analyses

The GraphPad Prism software (version 8.3.0, GraphPad Software Inc, La Jolla, CA, USA) was used for all statistical analyses. The normality of the distribution for all numerical variables was evaluated using Shapiro-Wilk tests, and homogeneity of variance was assessed by *F* tests. Numerical variables are presented as means ± standard deviations or medians (Q1–Q3). Group differences were assessed using an independent *t*-test for normally distributed and homogeneous variance data, otherwise, the Mann-Whitney test was used. Comparisons of categorical variables were analyzed using chi-square and Fisher’s exact tests.

The structural and [^18^F]-FDG PET data were subjected to voxel-wised whole-brain two-sample t-tests based on the framework of a general linear model (GLM) in SPM12, with age and sex as covariates. Brain regions with significant volume and FDG changes were determined using a voxel threshold of *p* < 0.05 (familywise error [FWE]-corrected). We then conducted the atlas-based ROI analysis of the PET images to extract the regional SUVRs of the relays in limbic CSTC circuit for further correlation analyses. To compare metabolic connectivity between groups, we used non-parametric permutation tests with 5000 permutations to determine significance. The *p*-values were calculated as the fraction of the difference in distribution values that exceeded the difference value between the actual groups.

For the bvFTD group, we performed a Pearson’s correlation between the [^18^F]-FDG SUVR of the relays in the limbic CSTC circuit and neuropsychiatric assessment scores using a threshold of *p* < 0.05 (false discovery rate [FDR]-corrected). Furthermore, multivariable linear regression analyses were performed to examine whether the severity of behavior disruption (FBI total score, FBI apathy, FBI disinhibition) was associated with involvement of limbic striatum covarying out the general cognition (MMSE) or disease severity (FTLD-CDR). For all analyses, a *p*-value < 0.05 indicated statistical significance.

## Results

### Demographic features of the subjects

Demographic, cognitive, and behavioral features of the bvFTD patients, controls, asymptomatic carriers, and noncarriers are presented in Table 1. Asymptomatic MAPT carriers exhibited no symptoms or signs of any kind, including cognitive and motor impairment. In addition, none of the bvFTD patients displayed any symptoms or signs of motor disturbance. There were significant differences in MMSE, MoCA, FTLD-CDR, AVLT, BNT, and FBI scores between bvFTD patients and controls, but not between asymptomatic *MAPT* mutation carriers and noncarriers. The mean (standard deviation) estimated years from the symptom onset was 8.33 (1.875) in the *MAPT* mutation carrier group with a range of 4 to 13 years.

**Table 1:** Demographic and neuropsychiatric assessment data

	BvFTD patients (*n* = 33)	Controls (*n* =33)	Asymptomatic MAPT carriers (*n* = 6)	Noncarriers in the family (*n* = 12)	*P-*value bvFTD patients vs controls	*P-*value MAPT carriers vs noncarriers
Age	58.94 ± 9.87	55.82±10.05	49.00 ± 3.90	42.25 ± 9.21	0.20^a^	0.11^a^
Sex (male/female)	16/17	15/18	3/3	7/5	0.99^c^	0.99^c^
Years of education	10.62 ± 4.64	11.31 ±3.47	8.67 ± 0.52	10.55 ± 3.80	0.52^a^	0.25^a^
MMSE	16.52 ± 6.87	28.65 ± 2.07	28.67 ± 0.82	28.36 ± 2.06	<0.0001^b^	0.74^a^
MoCA	10.15 ± 6.25	26.06 ± 3.43	26.50 ± 1.23	26.18 ± 3.06	<0.0001^b^	0.81^a^
FTLD-CDR	10.98 ± 4.46	0 ± 0	0 ± 0	0 ± 0	<0.0001^b^	-
**Memory**						
AVLT: immediate recall	7.70 ± 7.38	23.81 ± 5.59	23.83 ± 2.32	26.00 ± 7.32	<0.0001^a^	0.50^a^
AVLT: delayed recall	1.35 ± 2.53	8.79 ± 2.98	8.67 ± 2.25	9.27 ± 3.16	<0.0001^a^	0.69^a^
**Executive function**						
TMT-A	111.60±39.95	50.48 ± 24.94	44.33 ± 14.98	42.00 ±18.75	<0.0001^b^	0.79^a^
TMT-B	247.80±82.95	88.44 ± 62.59	85.17 ± 35.37	63.67±31.04	<0.0001^a^	0.19^a^
**Language**						
BNT	11.63 ± 6.39	24.96 ± 3.83	25.00 ± 1.00	24.90 ± 2.08	<0.0001^b^	0.92^a^
**Behavior features**						
FBI total score	26.87 ± 14.94	1.67 ± 4.08	0 (0–6)	0 (0–2)	<0.0001^b^	0.84^b^
FBI apathy	17.58 ± 9.02	0.83 ± 2.04	0 (0–4)	0 (0–0)	<0.0001^b^	0.40^b^
FBI disinhibition	9.29 ± 7.88	0.83 ± 2.04	0 (0–2)	0 (0–1.5)	<0.001^b^	0.99^b^

Data are presented as means ± the standard deviations or medians (Q1–Q3)

*AVLT* auditory verbal learning test, *BNT* Boston Naming Test, *FTLD-CDR* Frontotemporal Lobar Degeneration-Clinical Dementia Rating, *FBI* Frontal Behavioral Inventory, *MMSE* Mini-Mental State Examination, *MoCA* Montreal Cognitive Assessment

^a^Based on unpaired *t*-tests

^b^Based on Mann-Whitney tests

^c^Based on Pearson’s chi-squared test

### Alteration in each striatal subregion

Compared with controls, bvFTD patients showed a significantly lower GM volume in the striatal limbic subregion, the executive subregion, the rostral motor subregion, and the caudal motor subregion, but not in the parietal subregion, occipital subregion, or temporal subregion, as shown in Supplementary Tables 1 and 2. No gray matter loss was identified in asymptomatic MAPT carriers compared with non-carriers based on a voxel threshold of *p* < 0.05 (FWE-corrected).

Even though bvFTD patients did not exhibit any symptoms or signs of motor disturbance, when compared to controls, they showed gray matter loss and hypometabolism in bilateral motor subregions of the striatum, including the rostral-motor and caudal-motor subregion. Asymptomatic MAPT carriers and noncarriers did not significantly differ in GM volume and FDG uptake of bilateral motor subregions. These results are presented in Supplementary Tables 1 and 2 and Supplementary Figure 1.

### [^18^F]-FDG uptake in the relays of the limbic CSTC circuit

Bilateral hypometabolism was significantly more pronounced in bvFTD patients than in healthy controls in the relays within the limbic CSTC circuit, which included the vmPFC, OFC, rectus gyrus, ACC, thalamus, and limbic striatum (*p* < 0.0001; Supplementary Table 3). However, asymptomatic MAPT carriers and noncarriers did not significantly differ in FDG uptake of the above relays within the limbic CSTC circuit.

### Correlations between the limbic CSTC circuit and neuropsychological features

As shown in Fig. 1B and Supplementary Table 4, in the bvFTD group, SUVR values of the limbic striatum were negatively correlated with the FBI total score, disinhibition subscale score, apathy subscale score, and FTLD-CDR sum of boxes scale.

Similarly, significant diffusion associations were present between the SUVR values of the vmPFC, OFC, rectus gyrus, and ACC and the FBI total score, disinhibition subscale score, apathy subscale score, and FTLD-CDR sum of boxes scale score, as shown in Supplementary Table 4 and Supplementary Figures 2 and 3.

Multivariable linear regression analyses revealed that the FBI total score was significantly correlated with SUVR of the bilateral limbic striatum when the models were adjusted by MMSE. SUVR of the bilateral limbic striatum and FBI disinhibition subscale showed a trend of correlation, as did SUVR of the right limbic striatum and FBI apathy subscale. In addition, the FBI disinhibition subscale score was significantly correlated with SUVR of the bilateral limbic striatum when the models were adjusted by FTLD-CDR. The results were present in Supplementary Tables 5 and 6.

### Metabolic connectivity in the limbic CSTC circuit

**Fig. 2:**
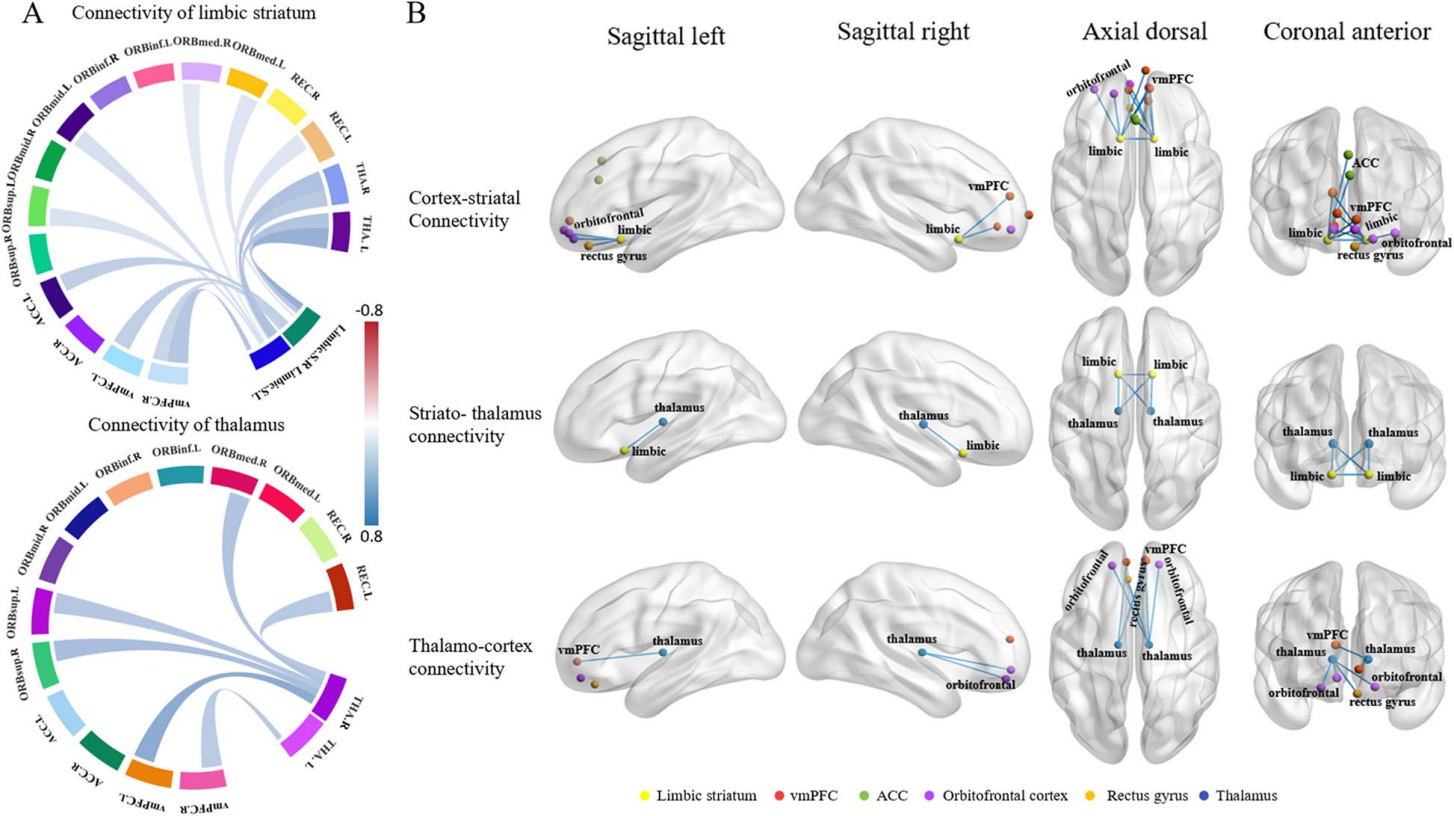
Metabolic connectivity within the limbic CSTC circuits in bvFTD patients. **A** Connectivity weights in bvFTD patients compared with controls. In bvFTD patients, extensively decreased metabolic connectivity between the major components of limbic CSTC circuits was observed. **B** Connectomes projected onto a 3D brain template. Enhanced metabolic connections are represented by red lines, and weakened metabolic connections are represented by blue lines. Abbreviations: vmPFC, ventromedial prefrontal cortex; ACC, anterior cingulate cortex

Compared with the controls, bvFTD patients showed the decreased metabolic connectivity within the CSTC circuit, including connectivity between the vmPFC, OFC, rectus gyrus, ACC, and the limbic striatum, between the limbic striatum and the thalamus, as well as between the thalamus and the prefrontal cortex (vmPFC, OFC, rectus gyrus, ACC), as shown in Fig. 2.

**Fig. 3:**
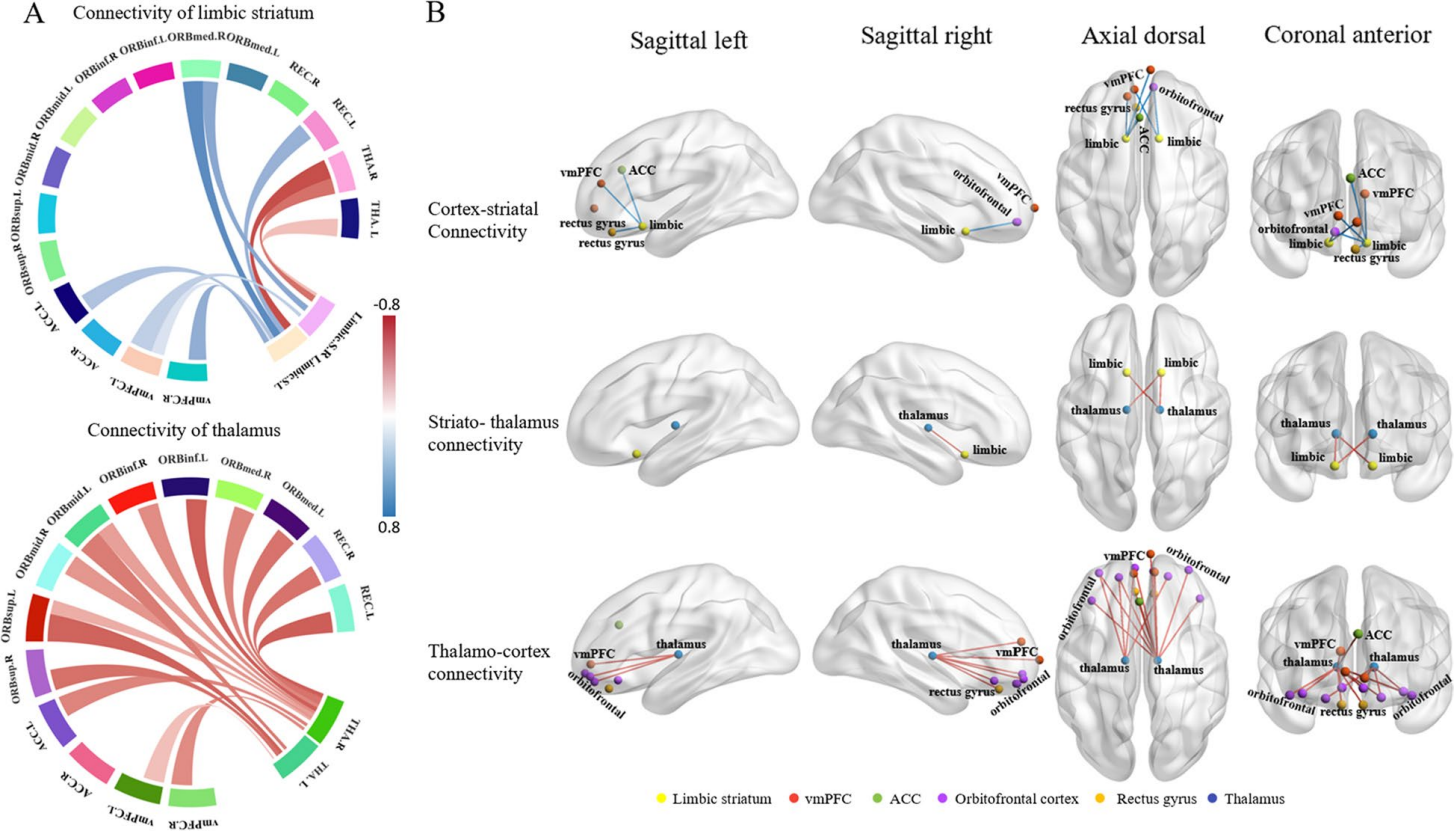
Metabolic connectivity within the limbic CSTC circuits in asymptomatic MAPT mutation carriers. **A** Connectivity weights in asymptomatic mutation carriers compared with noncarriers. Asymptomatic mutation carriers showed the decreased fronto-striatal connectivity and compensatory increased striato-thalamo and thalamo-cortical connection within limbic CSTC circuits. **B** Connectomes projected onto a 3D brain template. Enhanced metabolic connections are represented by red lines, and weakened metabolic connections are represented by blue lines. Abbreviations: vmPFC, ventromedial prefrontal cortex; ACC, anterior cingulate cortex

In asymptomatic *MAPT* carriers, the limbic subregions of the striatum showed weakened connections with vmPFC, OFC, rectus gyrus, and ACC, but enhanced connectivity with the thalamus compared with non-carriers. Accordingly, the thalamus had enhanced connections with the vmPFC, OFC, rectus gyrus, and ACC (Fig. 3).

## Discussion

We found altered connectivity within the limbic CSTC circuit in the bvFTD patients and presymptomatic *MAPT* mutation carriers, which, to the best of our knowledge, has not been empirically demonstrated previously. Specifically, extensive decreased connections were observed in the limbic CSTC circuit, which may be associated with more severe behavioral disturbances in bvFTD patients with both apathetic and disinhibition syndromes. In contrast to the connectivity patterns observed in bvFTD patients, the asymptomatic *MAPT* mutation carriers showed decreased frontostriatal connectivity and compensatory increases in striato-thalamic and thalamo-cortical connections in the limbic CSTC circuits, which may contribute to the absence of the symptom.

We revealed alterations in metabolic connectivity of the CSTC circuit in presymptomatic and symptomatic bvFTD, especially at the level of the limbic striatum associated with the behavior and psychiatric function, which has not been specifically investigated to date. Instead of using anatomically defined discrete striatal regions (nucleus accumbens, caudate nucleus, and putamen) as previously employed, we use a connectivity-based (CB) functional striatum atlas based on their distinct cortical connectivity profiles, which provides optimal subdivision to investigate specific functions of the striatum. We observed reduced metabolic connectivity within these circuits in our bvFTD patients, which is consistent with previous findings of both metabolic and structural abnormalities within regions comprising the limbic CSTC circuits in frontotemporal dementia (FTD) [5, 24]. Furthermore, the functional connectivity patterns of the limbic CSTC circuits were altered during the presymptomatic stage of *MAPT* mutation carriers at risk of genetic bvFTD. The above findings support previous studies that suggest that FTD is a more complex disease involving the dysfunctions in the cortical and subcortical transmission rather than dysfunctions in cortico-cortical transmission alone [5, 7, 24]. Furthermore, our study highlights that dysfunctions of cortico-subcortical transmission may occur during a very early stage of the disease. Notably, our study provided an opportunity to characterize in vivo changes in the limbic CSTC circuits from the preclinical to the symptomatic stage of bvFTD, which has not yet been experimentally established in previous studies. Taken together, the aberrance of the limbic CSTC circuit may be a sensitive and useful method for recognizing the presymptomatic stage of bvFTD and tracking disease progression.

In addition to disruption in behavior, personality, and social cognition, extensive cognitive impairment, including memory, execution, and language, were detected in bvFTD patients in this study. According to the most recent bvFTD diagnostic criteria, the neuropsychological profile includes executive dysfunction in the context of relatively preserved episodic memory and visuospatial skills [19]. Interestingly, significant memory impairment was found in our bvFTD patients, which was in line with previous studies that included pathologically proven patients with bvFTD, implying that memory deficit may be associated with the disease progression or with the fact that a portion of bvFTD does have amnestic disturbance [25–27].

Our FDG-PET findings showed that the limbic striatum which serves as the key subcortical relay of the limbic CSTC circuit is involved in bvFTD symptomatology, particularly in the behavioral and psychiatric disturbances of both apathetic and disinhibition syndromes. The hypometabolism of the limbic striatum in bvFTD patients was associated with the severity of the disease, which is consistent with the previous studies of FTD that used morphological and diffusion imaging [5, 24]. Notably, the SUVR of the bilateral limbic striatum was correlated with behavioral disturbances, as measured by the FBI total score, disinhibition subscale score, and apathy subscale score, which has never been studied to date. Moreover, the correlation analysis revealed that the SUVR of the vmPFC, OFC, rectus gyrus, and thalamus, which were identified as relays of the limbic CSTC circuits, was linked significantly to behavioral disturbances. In summary, these findings imply that the involvement of the limbic CSTC circuits might contribute to the central features of bvFTD including behavioral and psychiatric symptoms.

In both bvFTD patients and asymptomatic *MAPT* carriers, the frontostriatal connectivity in the limbic CTSC circuit was shown to be reduced. This finding is partly consistent with previous research that used DTI, which reported structural hypoconnectivity between the OFC and limbic striatum in bvFTD patients [5]. Interestingly, in contrast to the decreased metabolic connectivity observed in bvFTD patients, the asymptomatic MAPT mutation carriers showed increased connectivity between the limbic striatum and the thalamus, as well as between the thalamus and the frontal cortex within the limbic CTSC circuitry. This result suggested that increased connectivity in the CTSC circuitry during the very early stages of the disease may reflect compensatory or maladaptive remodeling, which then decreases as the disease progresses. Previous research has also discovered that the regional volumes of the striatum and thalamus connected to the medial prefrontal cortex in mild bvFTD patients were significantly larger than those in controls [5]. Thus, we speculate that the frontostriatal connectivity may be the initially involved in the asymptomatic *MAPT* mutation carriers, and enhanced connectivity between the other relays of the limbic CTSC circuit may reflect a compensatory process to help maintain normal function. In addition, asymptomatic MAPT carriers showed altered metabolic connectivity without corresponding hypometabolism in the relays of the limbic CSTC circuit, which is consistent with recent studies that reported that intracortical transmission deficits preceded the emergence of white matter lesions, structural brain atrophy, degeneration of neuronal synapses, and cognitive impairment [18, 28, 29]. Taken together, aberrant metabolic connectivity within the limbic CSTC circuit, albeit with different patterns, was present during both the presymptomatic and symptomatic disease stages of bvFTD and was possibly related to the underlying pathophysiological process, implying that the earliest involvement in the bvFTD continuum occur in the frontostriatal connectivity within the limbic CTSC circuit.

## Limitations

This study has several limitations. First, the results were limited by the small sample size, particularly for asymptomatic *MAPT* mutation carriers, because FTD families that carry the *MAPT* mutation are rare. Second, this was a cross-sectional study. We plan to conduct longitudinal studies with postmortem confirmation in the future in asymptomatic *MAPT* mutation carriers and symptomatic bvFTD patients. Third, the asymptomatic *MAPT* subjects and subjects with bvFTD were each matched to a control group, which reduced their comparability. Nevertheless, the groups were as close as possible in age, and age was included in all analyses as a covariate to minimize potential confounds. Additionally, from a connectivity perspective, the directionality of the fronto-striatum, striato-thalamic, and thalamo-frontal connectivity could not be separated by FDG-PET imaging, which may have confounded the correlations.

## Conclusion

Our FDG-derived metabolic connectivity study in bvFTD patients and asymptomatic *MAPT* mutation carriers revealed abnormal functional connectivity within the limbic CSTC circuits. This study provided clinically relevant insights into the features of the limbic CSTC circuit in bvFTD patients, and these disrupted metabolic connectivity patterns may be eventually be used as imaging biomarkers to detect the presymptomatic stage of bvFTD and monitor disease progression. In the future, larger but foremost longitudinal studies will be needed to confirm the aberrant connectivity patterns of the limbic CSTC circuits in a continuous spectrum of bvFTD and elucidate the sequential order of these potential markers during the evolution of the disease.

## Supplementary Information

**Additional file 1: Supplementary Table 1.** Spatial coordinates and peak values of the striatal subregion showing significant differences in gray matter volume between bvFTD patients and controls.

**Additional file 2: Supplementary Table 2.** Results of gray matter volume in each of the striatal subregions with significant differences between bvFTD patients and normal controls.

**Additional file 3: Supplementary Figure 1.** Metabolism in the striatal motor subregion in bvFTD patients and asymptomatic MAPT carriers.

**Additional file 4: Supplementary Table 3.** Results of [^18^F]-FDG uptake in the relays of the limbic CSTC circuit.

**Additional file 5: Supplementary Table 4.** Results of the partial Pearson’s correlation analysis between SUVR of relays in limbic CSTC circuits and neuropsychiatric scores.

**Additional file 6: Supplementary Figure 2.** Scatter plots of the significant correlations between the SUVR values of the vmPFC, ACC, and rectus gyrus and neuropsychiatric scores. In the bvFTD group, SUVR values of the vmPFC, rectus gyrus and ACC were negatively correlated with the FBI total score, disinhibition subscale score, apathy subscale score and FTLD-CDR score. There were no significant associations between hypometabolism in the vmPFC, rectus gyrus, and ACC and the MMSE of MoCA score. Regions and scatterplot colors, red: vmPFC, blue: rectus gyrus, green: ACC.

**Additional file 7: Supplementary Figure 3.** Scatter plots of the significant correlations between the SUVR value of the OFC and neuropsychiatric scores. In the bvFTD group, the SUVR values of the OFC was negatively correlated with the FBI total score, disinhibition subscale score, apathy subscale score, and FTLD-CDR score. No significant correlations were found between the SUVR value of the OFC and the MMSE or MoCA score.

**Additional file 8: Supplementary Table 5.** Relationship between FDG SUVR of limbic striatum and behavioural measures using multiple linear regression, adjusted by MMSE.

**Additional file 9: Supplementary Table 6.** Relationship between FDG SUVR of limbic striatum and behavioural measures using multiple linear regression, adjusted by FTLD-CDR.

## Data Availability

The datasets used and analyzed during the current study are available from the corresponding author on reasonable request.

## Abbreviations

ACC: Anterior cingulate cortex
CSTC: Cortico-striato-thalamic-cortical circuit
bvFTD: Behavioral variant frontotemporal dementia
FDG: Fluorodeoxyglucose
TMT: Trail-Making Test
vmPFC: Ventromedial prefrontal cortex

## References

[1] Wolters EE, Papma JM, Verfaillie SCJ, Visser D, Weltings E, Groot C (2021). [(18)F]Flortaucipir PET Across Various MAPT Mutations in Presymptomatic and Symptomatic Carriers. Neurology.

[2] Shpilyukova YA, Fedotova EY, Illarioshkin SN (2020). Genetic Diversity in Frontotemporal Dementia. Mol Biol.

[3] Liu L, Cui B, Chu M, Cui Y, Jing D, Li D (2021). The Frequency of Genetic Mutations Associated With Behavioral Variant Frontotemporal Dementia in Chinese Han Patients. Front Aging Neurosci.

[4] Panman JL, Jiskoot LC, Bouts M, Meeter LHH, van der Ende EL, Poos JM (2019). Gray and white matter changes in presymptomatic genetic frontotemporal dementia: a longitudinal MRI study. Neurobiol Aging.

[5] Jakabek D, Power BD, Macfarlane MD, Walterfang M, Velakoulis D, van Westen D (2018). Regional structural hypo- and hyperconnectivity of frontal-striatal and frontal-thalamic pathways in behavioral variant frontotemporal dementia. Hum Brain Mapp.

[6] Landin-Romero R, Kumfor F, Leyton CE, Irish M, Hodges JR, Piguet O (2017). Disease-specific patterns of cortical and subcortical degeneration in a longitudinal study of Alzheimer's disease and behavioural-variant frontotemporal dementia. Neuroimage..

[7] Bede P, Omer T, Finegan E, Chipika RH, Iyer PM, Doherty MA (2018). Connectivity-based characterisation of subcortical grey matter pathology in frontotemporal dementia and ALS: a multimodal neuroimaging study. Brain Imaging Behav.

[8] Halabi C, Halabi A, Dean DL, Wang PN, Boxer AL, Trojanowski JQ (2013). Patterns of striatal degeneration in frontotemporal dementia. Alzheimer Dis Assoc Disord.

[9] Tziortzi AC, Haber SN, Searle GE, Tsoumpas C, Long CJ, Shotbolt P (2014). Connectivity-based functional analysis of dopamine release in the striatum using diffusion-weighted MRI and positron emission tomography. Cereb Cortex.

[10] Aoki S, Smith JB, Li H, Yan X, Igarashi M, Coulon P, et al. An open cortico-basal ganglia loop allows limbic control over motor output via the nigrothalamic pathway. Elife. 2019;8. 10.7554/eLife.49995 Epub 2019/09/07. PubMed PMID: 31490123; PubMed Central PMCID: PMCPMC6731092. 10.7554/eLife.49995 PMC6731092 31490123

[11] Cox J, Witten IB (2019). Striatal circuits for reward learning and decision-making. Nat Rev Neurosci.

[12] Cummings JL (1993). Frontal-subcortical circuits and human behavior. Arch Neurol.

[13] Posner J, Marsh R, Maia TV, Peterson BS, Gruber A, Simpson HB (2014). Reduced functional connectivity within the limbic cortico-striato-thalamo-cortical loop in unmedicated adults with obsessive-compulsive disorder. Hum Brain Mapp.

[14] Kubler D, Schroll H, Buchert R, Kuhn AA (2017). Cognitive performance correlates with the degree of dopaminergic degeneration in the associative part of the striatum in non-demented Parkinson's patients. J Neural Transm (Vienna).

[15] Olm CA, McMillan CT, Irwin DJ, Van Deerlin VM, Cook PA, Gee JC (2018). Longitudinal structural gray matter and white matter MRI changes in presymptomatic progranulin mutation carriers. Neuroimage Clin.

[16] Popuri K, Dowds E, Beg MF, Balachandar R, Bhalla M, Jacova C (2018). Gray matter changes in asymptomatic C9orf72 and GRN mutation carriers. Neuroimage Clin.

[17] Jacova CHG, Tawankanjanachot I, Dinelle K, McCormick S, Gonzalez M, Lee H, Sengdy P, Bouchard-Kerr P, Baker M, Rademakers R, Sossi V, Stoessl AJ, Feldman HH, Mackenzie IR (2013). Anterior brain glucose hypometabolism predates dementia in progranulin mutation carriers. Neurology..

[18] Liu L, Chu M, Nie B, Liu L, Xie K, Cui Y, et al. Reconfigured metabolism brain network in asymptomatic microtubule-associated protein tau mutation carriers: a graph theoretical analysis. Alzheimer's Res Ther. 2022;14(1). 10.1186/s13195-022-01000-z. 10.1186/s13195-022-01000-z PMC8996677 35410286

[19] Rascovsky K, Hodges JR, Knopman D, Mendez MF, Kramer JH, Neuhaus J (2011). Sensitivity of revised diagnostic criteria for the behavioural variant of frontotemporal dementia. Brain.

[20] Levin CS, Maramraju SH, Khalighi MM, Deller TW, Delso G, Jansen F (2016). Design Features and Mutual Compatibility Studies of the Time-of-Flight PET Capable GE SIGNA PET/MR System. IEEE Trans Med Imaging.

[21] Huang S, Li J, Sun L, Ye J, Fleisher A, Wu T (2010). Learning brain connectivity of Alzheimer's disease by sparse inverse covariance estimation. NeuroImage..

[22] Dosenbach NU, Nardos B, Cohen AL, Fair DA, Power JD, Church JA (2010). Prediction of individual brain maturity using fMRI. Science.

[23] Tzourio-Mazoyer N, Landeau B, Papathanassiou D, Crivello F, Etard O, Delcroix N (2002). Automated anatomical labeling of activations in SPM using a macroscopic anatomical parcellation of the MNI MRI single-subject brain. Neuroimage..

[24] Bertoux M, O'Callaghan C, Flanagan E, Hodges JR, Hornberger M (2015). Fronto-Striatal Atrophy in Behavioral Variant Frontotemporal Dementia and Alzheimer's Disease. Front Neurol.

[25] Barker MS, Manoochehri M, Rizer SJ, Appleby BS, Brushaber D, Dev SI (2021). Recognition memory and divergent cognitive profiles in prodromal genetic frontotemporal dementia. Cortex..

[26] Graham A, Davies R, Xuereb J, Halliday G, Kril J, Creasey H (2005). Pathologically proven frontotemporal dementia presenting with severe amnesia. Brain..

[27] Poos JM, Jiskoot LC, Papma JM, van Swieten JC, van den Berg E (2018). Meta-analytic Review of Memory Impairment in Behavioral Variant Frontotemporal Dementia. J Int Neuropsychol Soc.

[28] Benussi A, Gazzina S, Premi E, Cosseddu M, Archetti S, Dell'Era V (2019). Clinical and biomarker changes in presymptomatic genetic frontotemporal dementia. Neurobiol Aging.

[29] Morbelli S, Drzezga A, Perneczky R, Frisoni GB, Caroli A, van Berckel BN (2012). Resting metabolic connectivity in prodromal Alzheimer's disease. A European Alzheimer Disease Consortium (EADC) project. Neurobiol Aging.

