# Supplementary material for: Altered metabolic connectivity within the limbic cortico-striato-thalamo-cortical circuit in presymptomatic and symptomatic behavioral variant frontotemporal dementia
Source: Alzheimers Res Ther. 2023 Jan 5;15:3. doi: 10.1186/s13195-022-01157-7 (PMC9814421; doi:10.1186/s13195-022-01157-7)
Supplement: Supplementary file 1 — Additional file 1: Supplementary Table 1. Spatial coordinates and peak values of the striatal subregion showing significant differences in gray matter volume between bvFTD patients and controls. [file 13195_2022_1157_MOESM1_ESM.docx]

Supplementary Table 1. Spatial coordinates and peak values of the striatal subregion showing significant differences in gray matter volume between bvFTD patients and controls

| Region | Side | Cluster Size | MNI coordinate | T value |
| --- | --- | --- | --- | --- |
| Limbic region | L | 877 | -12 15 -14 | -12.15 |
| Limbic region | R | 713 | 12 15 -14 | -10.15 |
| Executive subregion | L | 1898 | -12 13 7 | -10.76 |
| Executive subregion | R | 1548 | 12 13 7 | -9.35 |
| Rostral-motor subregion | L | 156 | -29 1 -1 | -9.00 |
| Rostral-motor subregion | R | 102 | 29 1 -1 | -6.77 |
| Caudal motor subregion | L | 320 | -26 -6 -1 | -9.71 |
| Caudal motor subregion | R | 119 | 26 -6 -1 | -6.85 |

All regions survived the most stringent whole-brain family-wise error (FEW) correction for multiple comparisons at p < 0.05 (minimum cluster size 100 voxels).

Abbreviations: MNI, Montreal Neurological Institute.
