# Supplementary material for: Altered metabolic connectivity within the limbic cortico-striato-thalamo-cortical circuit in presymptomatic and symptomatic behavioral variant frontotemporal dementia
Source: Alzheimers Res Ther. 2023 Jan 5;15:3. doi: 10.1186/s13195-022-01157-7 (PMC9814421; doi:10.1186/s13195-022-01157-7)
Supplement: Supplementary file 2 — Additional file 2: Supplementary Table 2. Results of gray matter volume in each of the striatal subregions with significant differences between bvFTD patients and normal controls. [file 13195_2022_1157_MOESM2_ESM.docx]

Supplementary Table 2. Results of gray matter volume in each of the striatal subregions with significant differences between bvFTD patients and normal controls.

|  | bvFTD patients  (n = 32) | controls  (n =33) | Asymptomatic MAPT carriers  (n = 6) | Noncarriers in family  (n = 12) | *p* value  bvFTD patients  vs controls | *p* value  MAPT carriers  vs noncarriers |
| --- | --- | --- | --- | --- | --- | --- |
| **GM Volume（10^-3^）** |  |  |  |  |  |  |
| Limbic region (left) | 1.08 ± 0.30 | 1.70± 0.13 | 1.72 ± 0.17 | 1.79 ±0.12 | <0.0001 | 0.33 |
| Limbic region (right) | 1.11 ± 0.26 | 1.67± 0.14 | 1.70 ± 0.15 | 1.73 ± 0.16 | <0.0001 | 0.65 |
| Executive region (left) | 1.04 ± 0.30 | 1.60 ± 0.20 | 1.55 ± 0.13 | 1.64 ± 0.11 | <0.0001 | 0.14 |
| Executive region (right) | 1.15± 0.27 | 1.63 ± 0.20 | 1.60 ± 0.15 | 1.65 ± 0.14 | <0.0001 | 0.45 |
| Rostral-motor subregion (left) | 1.15 ± 0.25 | 1.63± 0.17 | 1.60 ± 0.13 | 1.70 ±0.14 | <0.0001 | 0.07 |
| Rostral-motor subregion (right) | 1.29 ± 0.24 | 1.69± 0.17 | 1.63 ± 0.11 | 1.76 ± 0.16 | <0.0001 | 0.07 |
| Caudal-motor subregion (left) | 0.96 ± 0.18 | 1.31 ± 0.12 | 1.29 ± 0.24 | 1.21 ± 0.20 | <0.0001 | 0.46 |
| Caudal-motor subregion (right) | 0.96± 0.18 | 1.24 ± 0.13 | 1.27 ± 0.23 | 1.16 ± 0.16 | <0.0001 | 0.28 |
