# Supplementary figures and images for: Altered metabolic connectivity within the limbic cortico-striato-thalamo-cortical circuit in presymptomatic and symptomatic behavioral variant frontotemporal dementia
Source: Alzheimers Res Ther. 2023 Jan 5;15:3. doi: 10.1186/s13195-022-01157-7 (PMC9814421; doi:10.1186/s13195-022-01157-7)

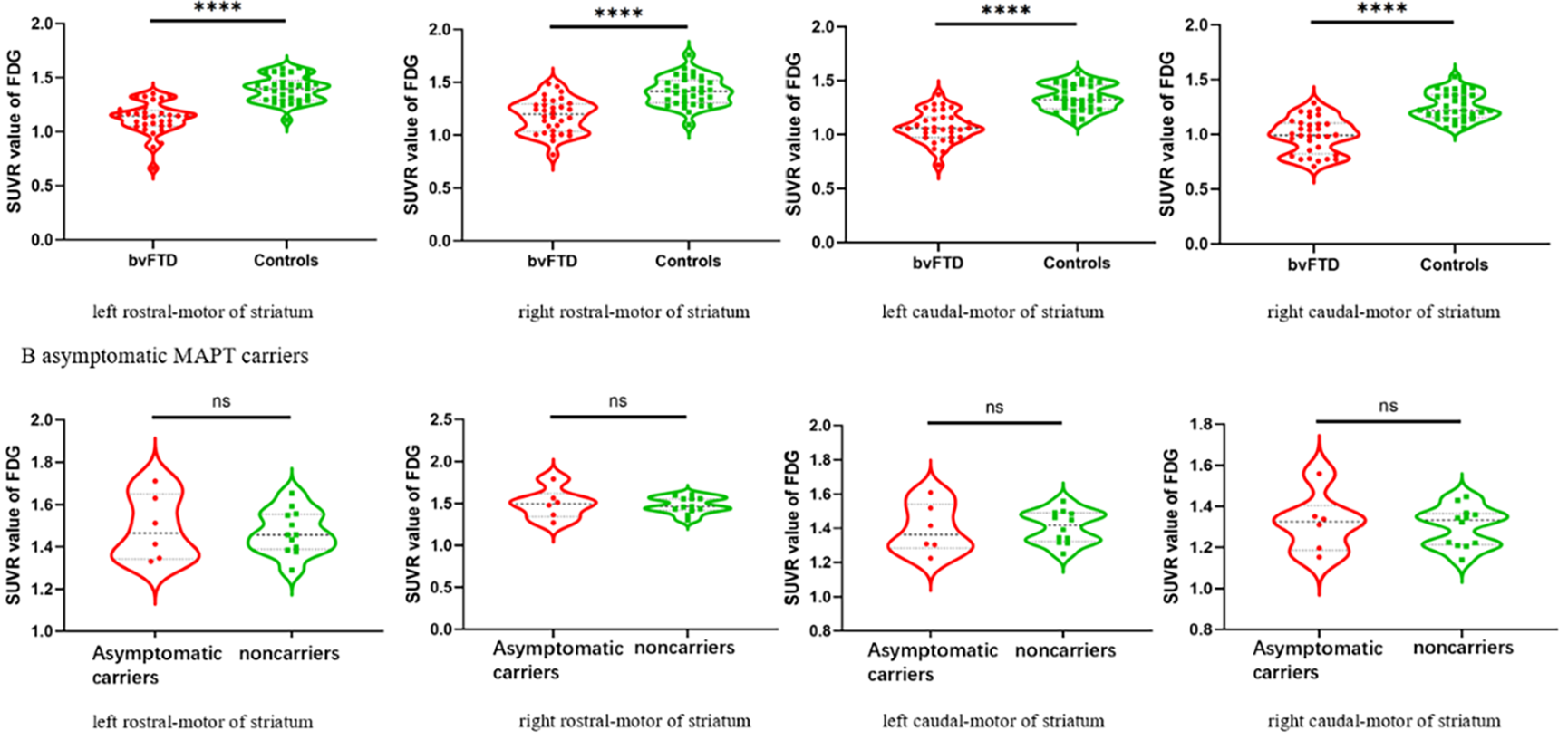

Supplement: Supplementary file 3 — Additional file 3: Supplementary Figure 1. Metabolism in the striatal motor subregion in bvFTD patients and asymptomatic MAPT carriers. [file 13195_2022_1157_MOESM3_ESM.tiff]

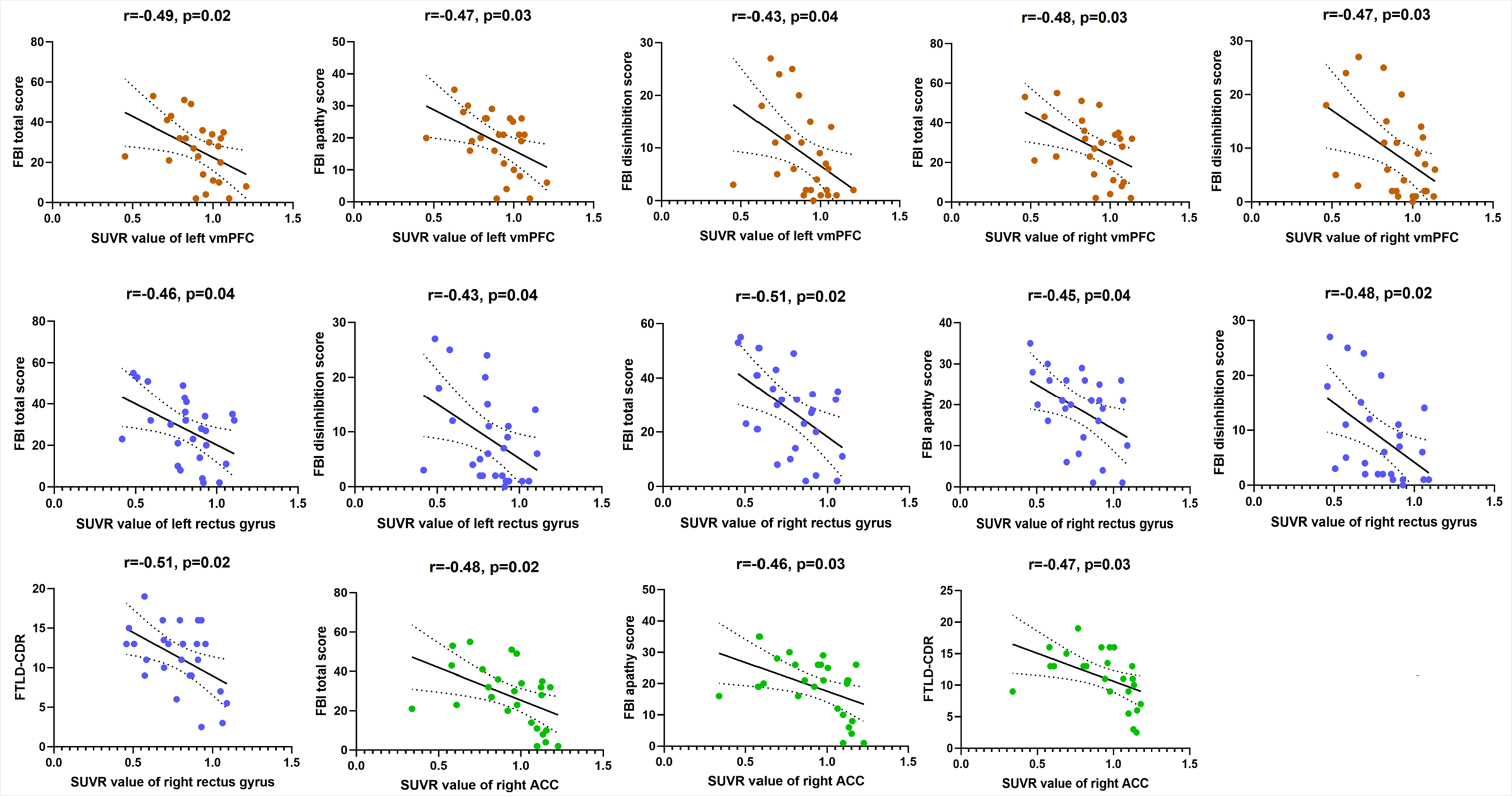

Supplement: Supplementary file 6 — Additional file 6: Supplementary Figure 2. Scatter plots of the significant correlations between the SUVR values of the vmPFC, ACC, and rectus gyrus and neuropsychiatric scores. In the bvFTD group, SUVR values of the vmPFC, rectus gyrus and ACC were negatively correlated with the FBI total score, disinhibition subscale score, apathy subscale score and FTLD-CDR score. There were no significant associations between hypometabolism in the vmPFC, rectus gyrus, and ACC and the MMSE of MoCA score. Regions and scatterplot colors, red: vmPFC, blue: rectus gyrus, green: ACC. [file 13195_2022_1157_MOESM6_ESM.tiff]

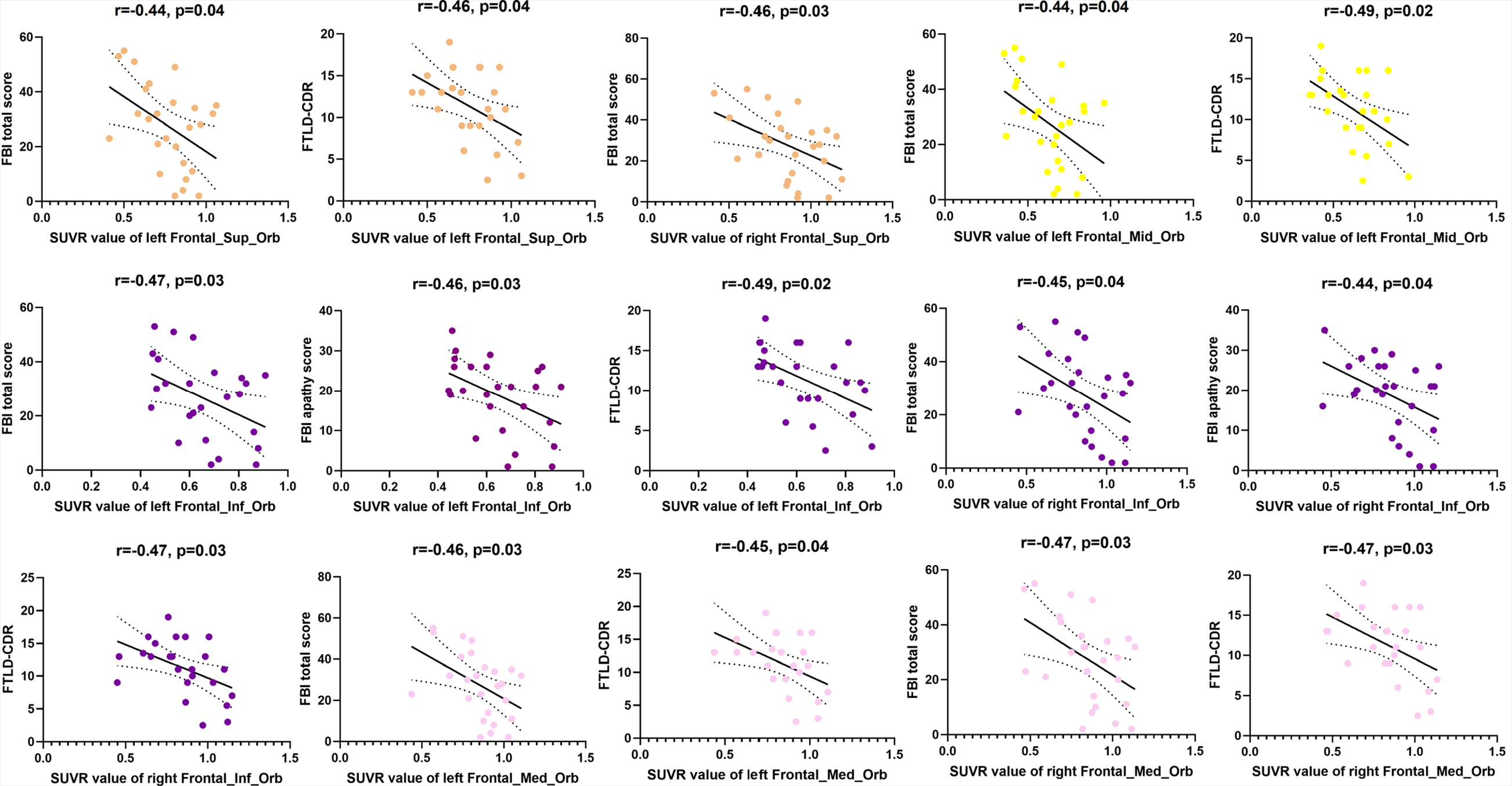

Supplement: Supplementary file 7 — Additional file 7: Supplementary Figure 3. Scatter plots of the significant correlations between the SUVR value of the OFC and neuropsychiatric scores. In the bvFTD group, the SUVR values of the OFC was negatively correlated with the FBI total score, disinhibition subscale score, apathy subscale score, and FTLD-CDR score. No significant correlations were found between the SUVR value of the OFC and the MMSE or MoCA score. [file 13195_2022_1157_MOESM7_ESM.tiff]
