# Supplementary material for: Altered metabolic connectivity within the limbic cortico-striato-thalamo-cortical circuit in presymptomatic and symptomatic behavioral variant frontotemporal dementia
Source: Alzheimers Res Ther. 2023 Jan 5;15:3. doi: 10.1186/s13195-022-01157-7 (PMC9814421; doi:10.1186/s13195-022-01157-7)
Supplement: Supplementary file 4 — Additional file 4: Supplementary Table 3. Results of [18F]-FDG uptake in the relays of the limbic CSTC circuit. [file 13195_2022_1157_MOESM4_ESM.docx]

Supplementary Table 3. Results of [^18^F]-FDG uptake in the relays of the limbic CSTC circuit.

| Region | Side | BvFTD patients  (n = 33) | Controls  (n =33) | Asymptomatic MAPT carriers  (n = 6) | Noncarriers in family  (n = 12) | *P-*value* bvFTD patients  vs controls | *P-*value^†^  MAPT carriers  vs noncarriers |
| --- | --- | --- | --- | --- | --- | --- | --- |
| **SUVR value of FDG** |  |  |  |  |  |  |  |
| Limbic region | L | 0.86 ± 0.16 | 1.12± 0.08 | 1.15 ± 0.08 | 1.12 ±0.08 | <0.0001 | 0.40 |
| Limbic region | R | 0.88 ± 0.17 | 1.11± 0.08 | 1.14 ± 0.09 | 1.12 ± 0.08 | <0.0001 | 0.57 |
| VmPFC | L | 0.90 ± 0.16 | 1.19± 0.09 | 1.22 ± 0.06 | 1.24 ± 0.12 | <0.0001 | 0.89 |
| VmPFC | R | 0.90 ± 0.18 | 1.21± 0.10 | 1.28 ± 0.12 | 1.29 ± 0.13 | <0.0001 | 0.96 |
| ACC | L | 0.94 ± 0.22 | 1.32± 0.12 | 1.50 ± 0.13 | 1.49 ± 0.14 | <0.0001 | 0.75 |
| ACC | R | 0.87 ± 0.23 | 1.37± 0.16 | 1.43 ± 0.12 | 1.42 ± 0.13 | <0.0001 | 0.75 |
| Frontal_Sup_Orb | L | 0.77 ± 0.17 | 1.05 ± 0.08 | 1.11 ± 0.09 | 1.08 ± 0.09 | <0.0001 | 0.49 |
| Frontal_Sup_Orb | R | 0.87 ± 0.20 | 1.11 ± 0.09 | 1.12 ± 0.08 | 1.14 ± 0.11 | <0.0001 | 0.68 |
| Frontal_Mid_Orb | L | 0.64 ± 0.16 | 0.93 ± 0.07 | 1.00 ± 0.10 | 0.94 ± 0.07 | <0.0001 | 0.43 |
| Frontal_Mid_Orb | R | 0.89 ± 0.21 | 1.11 ± 0.09 | 1.15 ± 0.11 | 1.14 ± 0.09 | <0.0001 | 0.62 |
| Frontal_Inf_Orb | L | 0.65 ± 0.15 | 0.94 ± 0.08 | 1.04 ± 0.08 | 0.99 ± 0.09 | <0.0001 | 0.33 |
| Frontal_Inf_Orb | R | 0.87 ± 0.20 | 1.13 ± 0.10 | 1.22 ± 0.12 | 1.20 ± 0.12 | <0.0001 | 0.68 |
| Frontal_Med_Orb | L | 0.85 ± 0.16 | 1.13 ± 0.09 | 1.18 ± 0.09 | 1.18 ± 0.10 | <0.0001 | 0.89 |
| Frontal_Med_Orb | R | 0.85 ± 0.19 | 1.20 ± 0.11 | 1.28 ± 0.12 | 1.29 ± 0.13 | <0.0001 | 0.89 |
| Rectus gyrus | L | 0.82 ± 0.18 | 1.12 ± 0.09 | 1.18 ± 0.09 | 1.17 ± 0.09 | <0.0001 | 0.75 |
| Rectus gyrus | R | 0.79 ± 0.19 | 1.15 ± 0.09 | 1.21 ± 0.12 | 1.21 ± 0.10 | <0.0001 | 0.99 |
| Thalamus | L | 1.02 ± 0.16 | 1.13 ± 0.12 | 1.08 ± 0.08 | 1.08 ± 0.09 | <0.01 | 0.99 |
| Thalamus | R | 1.03 ± 0.22 | 1.25 ± 0.13 | 1.24 ± 0.07 | 1.20 ± 0.11 | <0.0001 | 0.44 |

Data are presented as means ± the standard deviation.

*Two-sided p-values for continuous variables refer to unpaired t-tests.

^†^Two-sided *p*-values for continuous variables refer to Mann-Whitney tests.
