# Supplementary material for: Altered metabolic connectivity within the limbic cortico-striato-thalamo-cortical circuit in presymptomatic and symptomatic behavioral variant frontotemporal dementia
Source: Alzheimers Res Ther. 2023 Jan 5;15:3. doi: 10.1186/s13195-022-01157-7 (PMC9814421; doi:10.1186/s13195-022-01157-7)
Supplement: Supplementary file 5 — Additional file 5: Supplementary Table 4. Results of the partial Pearson’s correlation analysis between SUVR of relays in limbic CSTC circuits and neuropsychiatric scores. [file 13195_2022_1157_MOESM5_ESM.docx]

Supplementary Table 4. Results of the partial Pearson’s correlation analysis between SUVR of relays in limbic CSTC circuits and neuropsychiatric scores

| Region | Side | FBI  Total | FBI  Apathy | FBI Disinhibition | FTLD-CDR | MMSE | Moca |
| --- | --- | --- | --- | --- | --- | --- | --- |
| Limbic region | L | -0.5917^∗∗^ | -0.5153^∗∗^ | -0.5341^∗∗^ | -0.5443^∗∗^ | 0.4703 | 0.4863 |
| Limbic region | R | -0.6047^∗∗^ | -0.5345^∗∗^ | -0.5354^∗∗^ | -0.5430^∗∗^ | 0.4355 | 0.4056 |
| VmPFC | L | -0.4977^∗^ | -0.4664^∗^ | -0.4333^∗^ | -0.4340 | 0.3462 | 0.2652 |
| VmPFC | R | -0.4834^∗^ | -0.4097 | -0.4683^∗^ | -0.3635 | 0.3529 | 0.2079 |
| ACC | L | -0.2838 | -0.2889 | -0.2199 | -0.1784 | 0.1187 | 0.0531 |
| ACC | R | -0.4785^∗^ | -0.4556^∗^ | -0.4052 | -0.4736^∗^ | 0.3563 | 0.2455 |
| Frontal_Sup_Orb | L | -0.4425^∗^ | -0.4063 | -0.3932 | -0.4615^∗^ | 0.2469 | 0.2387 |
| Frontal_Sup_Orb | R | -0.4605^∗^ | -0.4291 | -0.4021 | -0.4196 | 0.2310 | 0.1502 |
| Frontal_Mid_Orb | L | -0.4401^∗^ | -0.3967 | -0.3996 | -0.4948^∗^ | 0.3525 | 0.3446 |
| Frontal_Mid_Orb | R | -0.3847 | -0.3634 | -0.3312 | -0.3779 | 0.2471 | 0.1388 |
| Frontal_Inf_Orb | L | -0.4742^∗^ | -0.4611^∗^ | -0.3932 | -0.4898^∗^ | 0.1844 | 0.2333 |
| Frontal_Inf_Orb | R | -0.4459^∗^ | -0.4385^∗^ | -0.3612 | -0.4697^∗^ | 0.2719 | 0.1788 |
| Frontal_Med_Orb | L | -0.4609^∗^ | -0.4210 | -0.4131 | -0.4491^∗^ | 0.2494 | 0.2072 |
| Frontal_Med_Orb | R | -0.4695^∗^ | -0.4206 | -0.4309 | -0.4658^∗^ | 0.2784 | 0.2069 |
| Rectus gyrus | L | -0.4570^∗^ | -0.3923 | -0.4346^∗^ | -0.4158 | 0.2168 | 0.2039 |
| Rectus gyrus | R | -0.5126^∗^ | -0.4454^∗^ | -0.4826^∗^ | -0.5060^∗^ | 0.2616 | 0.2303 |
| Thalamus | L | -0.2315 | -0.1991 | -0.2187 | -0.2400 | 0.1590 | 0.0258 |
| Thalamus | R | -0.0677 | 0.0487 | -0.1841 | -0.0264 | -0.2101 | -0.1711 |

^*^of statistical significance after false discovery rate [FDR]-corrected (p < 0.05);

^**^of statistical significance after false discovery rate [FDR]-corrected (p < 0.01).

Abbreviations: vmPFC, ventromedial prefrontal cortex; ACC, anterior cingulate cortex
