# Supplementary material for: Altered metabolic connectivity within the limbic cortico-striato-thalamo-cortical circuit in presymptomatic and symptomatic behavioral variant frontotemporal dementia
Source: Alzheimers Res Ther. 2023 Jan 5;15:3. doi: 10.1186/s13195-022-01157-7 (PMC9814421; doi:10.1186/s13195-022-01157-7)
Supplement: Supplementary file 8 — Additional file 8: Supplementary Table 5. Relationship between FDG SUVR of limbic striatum and behavioural measures using multiple linear regression, adjusted by MMSE. [file 13195_2022_1157_MOESM8_ESM.docx]

Supplementary Table 5. Relationship between FDG SUVR of limbic striatum and behavioural measures using multiple linear regression, adjusted by MMSE

| Variables | B | β | 95%CI | t | *P* value |
| --- | --- | --- | --- | --- | --- |
| Left limbic striatum |  |  |  |  |  |
| FBI total score | -39.988 | -0.414 | (-79.804, -0.172) | -2.078 | 0.04 |
| FBI apathy | -20.898 | -0.333 | (-46.185, 4.389) | -1.710 | 0.101 |
| FBI disinhibition | -19.090 | -0.42 | (-39.397, 1.217) | -1.945 | 0.064 |
| Right limbic striatum |  |  |  |  |  |
| FBI total score | -40.172 | -0.441 | (-76.325, -4.019) | -2.299 | 0.031 |
| FBI apathy | -22.576 | -0.381 | (-45.388, 0.237) | -2.047 | 0.052 |
| FBI disinhibition | -17.596 | -0.410 | (-36.366, 1.173) | -1.939 | 0.065 |
