# Supplementary material for: Altered metabolic connectivity within the limbic cortico-striato-thalamo-cortical circuit in presymptomatic and symptomatic behavioral variant frontotemporal dementia
Source: Alzheimers Res Ther. 2023 Jan 5;15:3. doi: 10.1186/s13195-022-01157-7 (PMC9814421; doi:10.1186/s13195-022-01157-7)
Supplement: Supplementary file 9 — Additional file 9: Supplementary Table 6. Relationship between FDG SUVR of limbic striatum and behavioural measures using multiple linear regression, adjusted by FTLD-CDR. [file 13195_2022_1157_MOESM9_ESM.docx]

Supplementary Table 6. Relationship between FDG SUVR of limbic striatum and behavioural measures using multiple linear regression, adjusted by FTLD-CDR

| Variables | B | β | 95%CI | t | *P* value |
| --- | --- | --- | --- | --- | --- |
| Left limbic striatum |  |  |  |  |  |
| FBI total score | -28.551 | -0.307 | (-65.801, 8.699) | -1.579 | 0.127 |
| FBI apathy | -13.449 | -0.248 | (-34.474, 7.576) | -1.315 | 0.2 |
| FBI disinhibition | -22.400 | -0.456 | (-42.526, -2.273) | -2.288 | 0.031 |
| Right limbic striatum |  |  |  |  |  |
| FBI total score | -28.364 | -0.306 | (-65.355, 8.627) | -1.579 | 0.127 |
| FBI apathy | -13.466 | -0.242 | (-35.089, 8.156) | -1.28 | 0.212 |
| FBI disinhibition | -21.714 | -0.43 | (-42.603, -0.824) | -2.137 | 0.04 |
